# Visualizing polymeric bioresorbable scaffolds with three-dimensional image reconstruction using contrast-enhanced micro-computed tomography

**DOI:** 10.1007/s10554-016-1049-z

**Published:** 2016-12-30

**Authors:** Sheng Tu, Fudong Hu, Wei Cai, Liyan Xiao, Linlin Zhang, Hong Zheng, Qiong Jiang, Lianglong Chen

**Affiliations:** 1 Department of Cardiology, Fujian Medical University Union Hospital, Fujian Institute of Coronary Heart Disease, Fuzhou, Fujian People’s Republic of China; 2 Department of Cardiology, The First Affiliated Hospital of Zhengzhou University, Zhengzhou, Henan People’s Republic of China; 3 Intensive Care Unit, The Second Affiliated Hospital of Fujian Medical University, Quanzhou, Fujian People’s Republic of China

**Keywords:** Bioresorbable scaffolds, Micro-computed tomography, Contrast medium, Three-dimensional reconstruction

## Abstract

There are no previous studies showing how to visualize polymeric bioresorbable scaffolds (BRSs) by micro-computed tomography (mCT). There are no previous studies showing how to visualize polymeric bioresorbable scaffolds (BRSs) by micro-computed tomography (mCT). This study aimed to explore the feasibility of detecting polymeric BRS with 3-dimensional reconstruction of BRS images by contrast-enhanced mCT and to determine the optimal imaging settings. BRSs, made of poly-l-lactic acid (PLLA), were implanted in coronary bifurcation models. Five treatments were conducted to examine an optimal condition for imaging BRSs: Baseline treatment, samples were filled with normal saline and scanned with mCT immediately; Treatment-1, -2, -3 and -4, samples were filled with contrast medium and scanned with mCT immediately and 1, 2 and 3 h thereafter, corresponding to soaking time of contrast medium of 0, 1, 2 and 3 h. Compared to Baseline, mCT scanning completely discriminate the scaffold struts from the vascular lumen immediately after filling the samples with contrast agent but not from the vascular wall until the contrast agent soaking time was more than 2 h (Treatment-3 and -4). By setting 10–15 HU as a cut-point of CT values, the scaffold strut detectable rate at Baseline and Teatment-1, -2, -3 and -4 were 1.23 ± 0.31%, 1.65 ± 0.26%, 58.14 ± 12.84%, 97.97 ± 1.43% and 98.90 ± 0.38%, respectively (Treatment-3 vs. Treatment-2, p < 0.01); meanwhile, the success rate of 3D BRS reconstruction with high quality images at Baseline and Teatment-1, -2, -3 and -4 were 1.23%, 1.65%, 58.14%, 97.97% and 98.90%, respectively (Treatment-3 vs. Treatment-2, p < 0.01). In conclusions, reconstruction of 3D BRS images is technically feasible by contrast-enhanced mCT and soaking time of contrast agent for more than 2 h is necessary for complete separation of scaffold struts from the surrounding structures in the phantom samples.

**Electronic supplementary material:**

The online version of this article (doi:10.1007/s10554-016-1049-z) contains supplementary material, which is available to authorized users.

## Introduction

Bioresorbable scaffolds (BRSs), as the newest generation of intracoronary stents, have shown an attractive prospect due to their superiority over the conventional metal-based stents [1–4]. However, until BRSs can be safely used for patients with complex lesions (tortuous, calcified or bifurcated lesions), it is necessary to strictly examine their maneuverability (deliverability, trackability), and more importantly, mechanical properties (expandability, durability, anti-fracture ability) [5, 6]. Hence, bench testing is necessary while micro-computed tomography (mCT) is deemed to be an essential imaging tool for visualization of BRS configurations. However, the polymeric BRSs used currently are invisible directly by mCT. Up to date, there are no adequate studies showing how to image BRS by using mCT, particularly for the optimal conditions for initial acquisition of the X-ray raw data and subsequent reconstruction of 2- or 3-dimensional (2D/3D) images albeit such images have been shown in several previous in vitro studies [7].

In this study, we explore the feasibility of imaging polymeric BRS with 3-dimensional reconstruction of BRS images by contrast-enhanced mCT and to determine the optimal imaging settings in bench testing.

## Materials and methods

### Materials

Polymeric BRS (Neovas™, LePu Medical, Beijing, China), a poly-l-lactic acid (PLLA) scaffold, was served as testing scaffold and Ultravist (370 mg/ml, iopromide, Bayer Pharma AG), an iodinated medium, as the contrast agent.

A coronary artery bifurcation model, made of polyvinyl alcohol according to Murray’s law, was adopted for bench testing, which has the distal bifurcation angle (DBA) of 60° and branch diameter difference (BDD) of 0.50 mm. The bifurcation model and BRS were incubated in a thermostat water bath of 37 °Cduring scaffold deployment.

### Experimental protocol

To determine an optimal condition for contrast-enhanced mCT imaging, 15 phantom samples (scaffolds implanted in the bifurcated model) received the following treatments: Baseline or Control treatment, samples filled with normal saline and scanned with mCT; Treatment-1, -2, -3 and -4, samples filled with contrast medium and scanned with mCT immediately and after 1, 2 and 3 h, corresponding to soaking time of contrast medium of 0, 1, 2 and 3 h.

### MCT scanning with raw data acquisition

CT scanning was performed using a mCT system (SkyScan 1176, Kontich, Belgium) to acquire a whole set of raw data along the entire length of a phantom sample. The scanning method and settings were: the sample was positioned on a rotary plate with 360° rotation at the speed of 0.36°/s, with a total of 800–1000 images recorded per sample. The X-ray parameter was set at 65 kV and 385 μA, and scanning with high spatial resolution of 18 μm.

### Analysis of raw data

The acquired raw data consisted of three components: phantom vascular wall (made of polyvinyl alcohol), vascular lumen (filled with contrast agent) and scaffold struts (rings and their connecting stems, made of PLLA). Usually, the phantom vascular wall was opacified partially (grey) with different degree dependently on the soaking time, vascular lumen opacified completely (bright) due to filling with contrast agent, and scaffold struts not opacified (dark) owing to PLLA resistance to contrast agent staining, resulting in extremely low radiopacity.

For quantitative analysis, we randomly selected a 6-mm segment from each phantom sample to measure the CT attenuation of the phantom vascular wall, vascular lumen and scaffold struts. By setting a cut-point of the CT value, the scaffold struts could be extracted from the phantom vascular wall and vascular lumen, and then the raw data composing of scaffold struts only was digitally converted into grey-scale images with DICOM format. Based on CT value between 10 and 15 HU as cut-points, scaffold strut detectable rate (SDR, %), calculated by detectable struts/total struts ×100, was optimal with almost no overlapping of CT values among the three components of phantom samples.

### 3D reconstruction of BRS

For offline 3D reconstruction, the grey-scale images (raw data composed of the scaffold struts only) were inputted into a computer installed with 3D reconstruction software (SkyScan 1176, Kontich, Belgium), the 3D BRS images could be automatically reconstructed with different quality.

The 3D image quality was graded according to the following criteria: (1) high quality, characterized by full visualization of whole BRS configuration with complete separation of all struts from surrounding structures; (2) suboptimal quality, by partial visualization of BRS configuration with missing some struts and incompletely separating some struts from surrounding structures; and (3) poor quality, by incomplete visualization of BRS configuration with missing many struts and incompletely separating many struts from surrounding structures. The reconstruction of 3D BRS images with high, suboptimal and poor quality was defined as success, partial success and failure, respectively.

### Statistical analysis

Data were analyzed with statistical software packages (SSPS 22.0; SSPS, Chicago, IL). Data were expressed as mean ± SD for continuous or frequency for categorical variables. Analysis of variance (ANOVA) was conducted for normally distributed continuous variables, followed by LSD test if significant; and Chi square or Fisher exact probability test for categorical variables as appropriate. A P value <0.05 was considered statistically significant.

## Results

### CT values and detectable struts in different settings

**Fig. 1:**
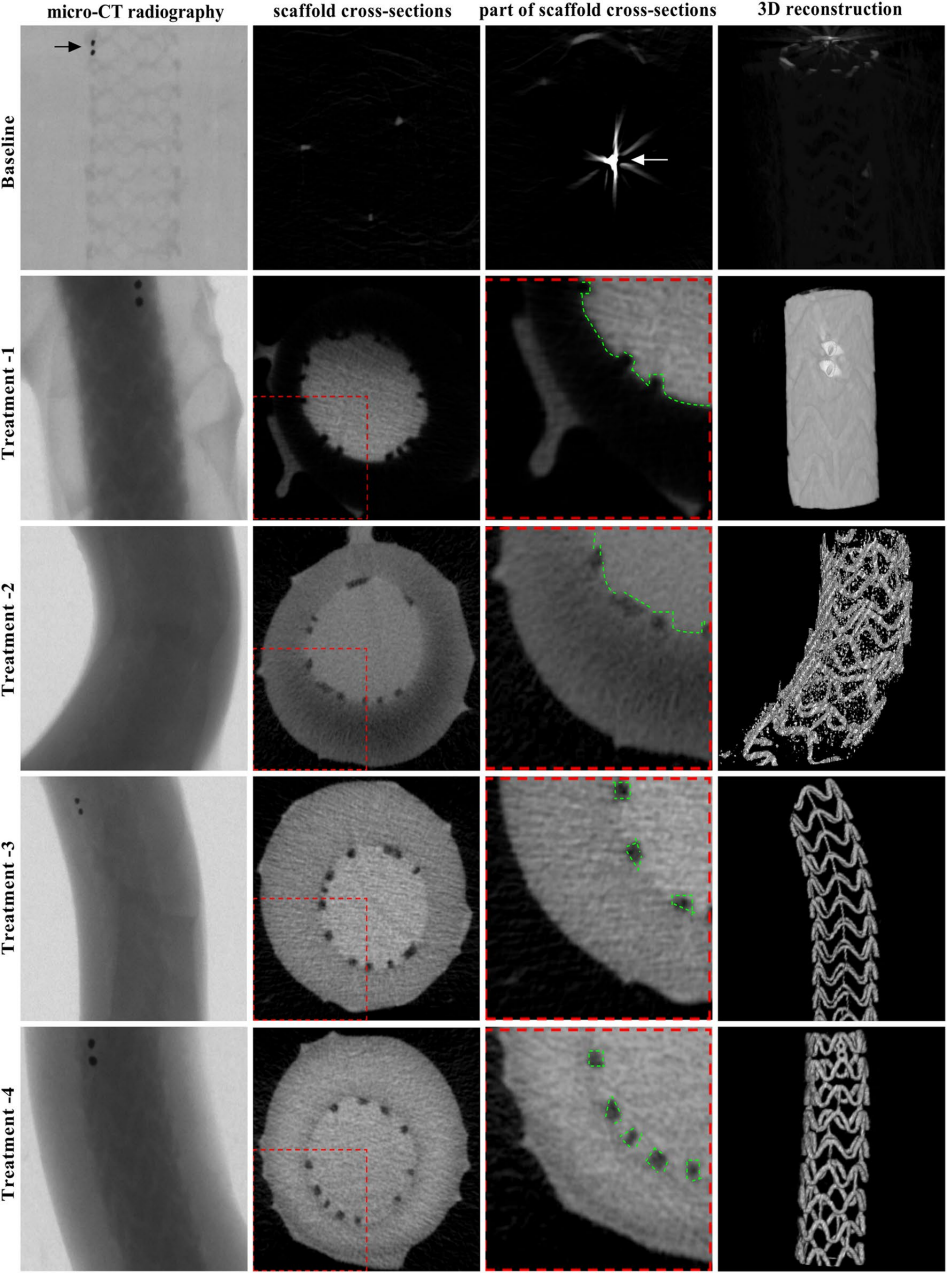
MCT raw data and reconstructed 3D images of BRS in different settings. The cross-sectional images (raw data) of a phantom sample acquired by mCT scanning and corresponding 3D reconstructed images of BRS in different treatments. At Baseline, mCT scanning was unable to distinguish the three components of the phantom sample with only gold markers being detected. Regardless of complete separation of the scaffold struts from the vascular lumen immediately after filling the lumen with contrast agent, mCT scanning could not clearly separate the struts from the vascular wall, failing to reconstruct a 3D BRS image in Treatment-1; and could only separate partial struts from the vascular wall, resulting in a suboptimal 3D BRS image with some contamination of the vascular wall signal in Treatment-2. Till to Treatment-3, mCT scanning was able to clearly separate the struts from the vascular wall, enabling to completely reconstruct 3D BRS images with high quality in Treatment-3 and -4

As shown in Table 1 and Fig. 1, the mean CT value was highest in the vascular lumen and lowest in the scaffold struts, which was constant independently on the soaking time of contrast agent; whilst the mean CT value was in-between in the vascular wall, which increased dependently on the soaking time. As a result, separation of the scaffold struts from the surrounding structures could be achieved as the soaking time was ≥2h as in the treatment 3–4, favoring distinguish of the scaffold struts from the vascular wall and vascular lumen along the entire length of a phantom sample.

**Table 1:** CT values and detectable struts in different contrast settings

	CT value (HU)			SDR (%)
	Vascular wall	Vascular lumen	Scaffold struts	
Baseline	3.06 ± 2.37	3.06 ± 2.19	3.13 ± 1.84	1.23 ± 0.31
Treatment-1	3.21 ± 2.24	231.73 ± 22.19*^,▲^	3.12 ± 2.35	1.65 ± 0.26
Treatment-2	107.91 ± 16.27*	228.72 ± 25.92*^,▲^	3.21 ± 2.39^∇,Δ^	58.14 ± 12.84*
Treatment-3	171.29 ± 16.97*^#^	232.15 ± 24.15*^,▲^	3.13 ± 2.46^∇,Δ^	97.97 ± 1.43*^,#^
Treatment-4	178.21 ± 16.62*^#^	233.92 ± 21.64*^,▲^	3.12 ± 2.41^∇,Δ^	98.90 ± 0.38*^,#^

Comparison of CT values among treatments: Treatment-1, -2, -3 and -4 vs. Baseline, *p < 0.01, respectively; Treatment-3 and -4 vs. Treatment-2, ^#^ *p* < 0.01, respectively

Comparison of CT values among 3-components of the phantom sample: versus vascular wall, ^∇^ *p* < 001; versus vascular lumen, ^Δ^ *p* < 001; versus vascular wall, ^▲^ *p* < 001

*SDR* scaffold detectable rate

### Reconstruction of 3D BRS images in different settings

The 3D BRS images with high quality could be obtained as the raw data were good enough in quality as shown in Fig. 1 and Movie 1–5. As listed in Table 2, mCT scanning was unable to reconstruct 3D BRS images at Baseline and in Treatment-1, and was able to partially reconstruct 3D BRS images in Treatment-2, with only partial success rate of 58.14%; while mCT scanning was able to completely reconstruct 3D BRS images with similarly higher success rate in Treatment-3 and -4.

**Table 2:** Quality of 3D reconstructed images in different contrast settings

	Image quality		
	Success (%)	Partial success (%)	Failure (%)
Baseline	1.23	0	98.77
Treatment-1	1.65	0	98.35
Treatment-2	0	58.14	41.86
Treatment-3	97.97*	2.03*	0^*^
Treatment-4	98.90*^,#^	1.10*^,#^	0*^,#^

Comparison of image quality among different contrast settings: Vs. Baseline, Treatment-1 and -2, *p < 0.01, respectively; vs. Treatment-3, ^#^ *p* > 0.05

### Discussion

MCT, with capability of directly visualizing metal stents, has been broadly adopted in assessment of stent performance ex vivo [8, 9]. However, most of BRSs currently available were made of PLLA that is invisible directly under mCT scanning. The present study was the first to systemically examine how to visualize BRS implanted in a bifurcated vascular phantom and then to reconstruct 3D BRS images in bench testing, thus offering a basic tool for exploring of BRS used in complex clinical scenarios and for optimizing the interventional procedures. Our major findings were: (a) soaking time of contrast agent for more than 2 h is necessary for complete separation of scaffold struts from the surrounding structures in the phantom samples; (b) reconstruction of 3D BRS images is technically feasible by contrast-enhanced mCT.

### Optimal setting for detecting struts and reconstructing 3D BRS images

The vascular bifurcation model used in the present study, made of polyvinyl alcohol, is permeable to iodized contrast media and the infiltrating amount or rate is mainly dependent on soaking time of contrast media, whereas BRSs currently used clinically, made of PLLA, is impermeable iodized contrast media or resistant to contrast media staining. Based on the rationale, it is possible to distinguish the scaffold struts from the phantom vascular wall and lumen with mCT scanning. Our study demonstrated that soaking time for 2 h or more is the best for complete separation of the scaffold struts from surrounding structures under mCT scanning and for full reconstruction of 3D BRS images with high quality as well.

In addition to the soaking time of the contrast agents, sorts of contrast agents, materials of polymeric BRS or vascular phantoms, and scanning settings including voltage/current, special and temporal resolutions may affect acquisition of the raw data, thus significantly influencing the optimal conditions for visualization of BRS struts and subsequent reconstruction of 3D BRS images [10, 11].

### Advantages of 3D BRS images

The studies using the metal stent platforms suggesting that the anatomic integrity of the vessel lumen plays a crucial role in the maintenance of normal hemodynamics such as local flow patterns and shear stresses. Accordingly, the presence of any abnormal geometric configuration (i.e., oval lumen shape, malapposed struts, localized stent deformation and so on) might be associated with turbulent flow patterns and driving pressure loss, impaired shear-stress pattern, abnormal platelet activation and even leading to severe clinical events or complications [12–16]. Usually, polymeric BRSs are much thicker and more fragile in physico-mechanical properties than metal stents. Despite that superiority of BRS in the treatment concept and clinical practice, its inferiority, for instances, larger profile, weaker support force, malapposed struts, localized stent distortion, ruptured struts or even scaffold collapse, and the associated abnormal hemodynamics, is prominent or even lethal that limits its broader clinical utility particularly for treatment of complex lesions. Accordingly, bench testing of BRS deployment with reconstruction of 3D BRS images using mCT scanning is essential to overwhelm the shortcoming associated with BRS and then to optimize stenting procedures in clinical practice.

**Fig. 2:**
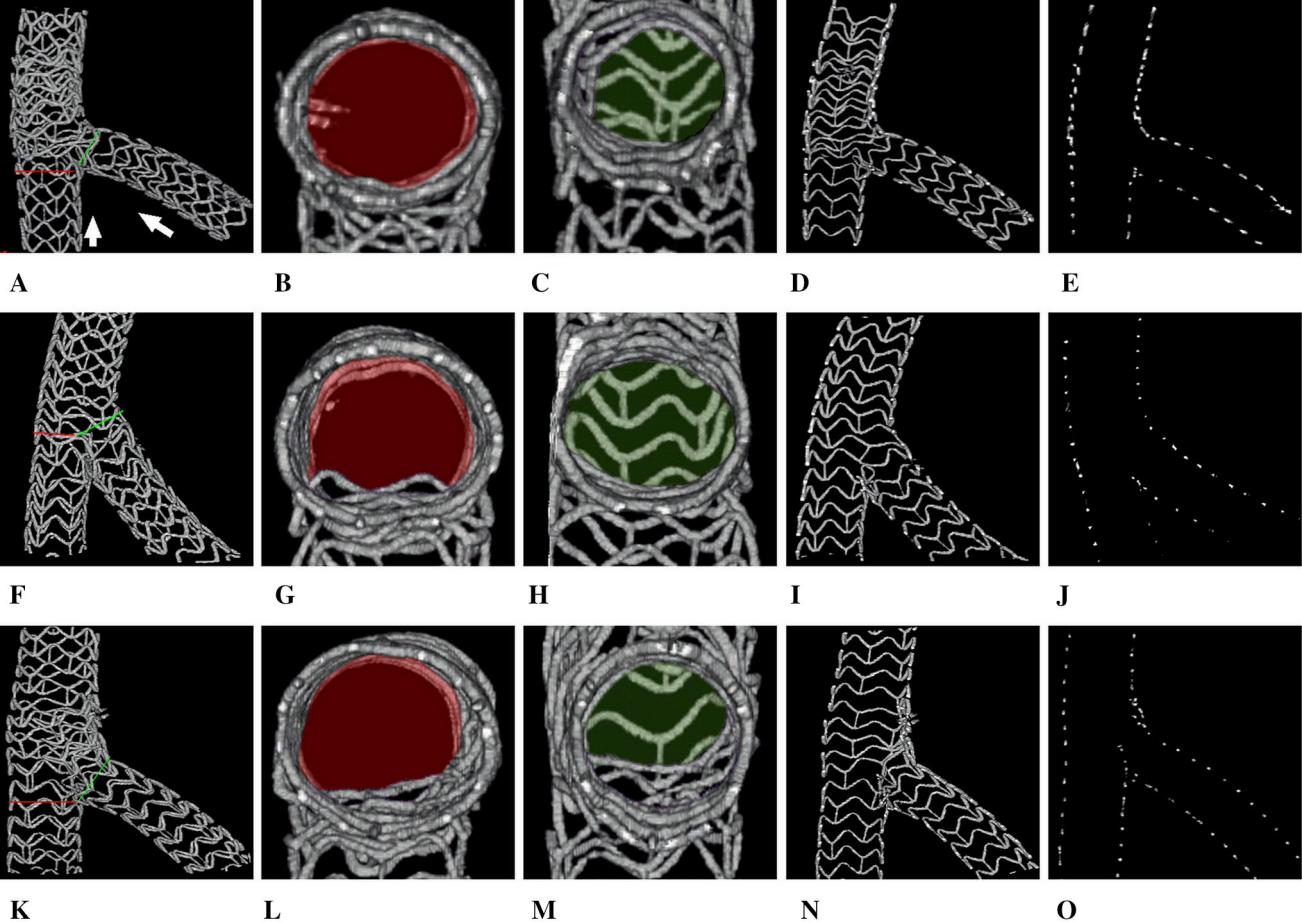
Display and application of 3D BRS images in bench testing. After acquisition of high quality raw data of BRSs by using contrast-enhanced mCT, 3D BRS images could be easily reconstructed and displaying in various formats: full 3D images (*1st row A, F, K*), transverse cutting images viewed distally to proximally, or vice versa, for inspection of MB ostium (*2nd row B, G, L*) and SB ostium (*3rd row C, H, M*), coronal cutting images for examination of bifurcated scaffold morphological features (e.g., scaffold expansion, coverage, overlapping, distortion, rupture and so on) (*4th row D, I, N*), and strut cross-sectional images (so called strut footprints) for accurately measuring key parameters of bifurcated scaffold morphology (e.g., scaffold luminal diameter and area, luminal symmetry, neocarina length and so on) (*5th row E, J, O*). Also, in this case, BRSs (LePu Medical, Beijing, China) was used to ex vivo emulate the three bifurcation stenting techniques: CULOTTE (*upper panels A, B, C, D, E*), TAP (*middle panels F, G, H, I, J*) and CRUSH (*lower panels K, L, M, N, O*) with clearly showing the morphological characteristics of different bifurcated stenting techniques by 3D reconstructed BRS images

After determination of the optimal settings for detection of scaffold struts by mCT and successful reconstruction of 3D BRS images by offline analysis, the scaffold configurations could be clearly visualized, enabling us to evaluation of scaffold expansion and coverage, strut malopposition, distortion and rupture. In present study, we systemically emulated various complex 2-stent techniques that used clinically for treatment of complex coronary bifurcation lesions, and successfully reconstructed 3D BRS images of each stenting technique, thereby affording essential information for morphological investigation and technical optimization of 2-scaffold techniques for bifurcation interventions (see Fig. 2 and Movie 6–8). Additionally, based on the high quality 3D BRS images, it is easy to examine the hemodynamic features associated with BRSs particularly in bifurcation interventions when combining with particle image velocimetry, echo particle image velocimetry or computational fluid dynamics techniques [17–21], thus offering another crucial information for optimization of 2-scaffold techniques from hemodynamic aspect.

### Limitations and future aspect

Regardless of establishment of an optimal setting for visualization and 3D-reconstruction of BRS, there are several limitations in the present study. Firstly, a suitable condition for detection BRS and reconstruction of its 3D images may be changeable dependently on not only the properties of phantom and BRS materials but also characteristics of contrast agents, mCT scanning setting, etc. Secondly, there remains possibility of suboptimal extraction of scaffold struts that may lead to suboptimal reconstruction of 3D BRS images. However, the limitations are avoidable and adjusting of settings may be necessary.

## Conclusions

The soaking time of contrast agent for more than 2 h is necessary for complete separation of scaffold struts from the surrounding structures in the phantom samples, and 3D reconstruction of BRS images is technically feasible by using contrast-enhanced mCT.

## Electronic supplementary material

Below is the link to the electronic supplementary material.

Supplementary material 1 (AVI 2668 KB)

Supplementary material 2 (AVI 4182 KB)

Supplementary material 3 (AVI 4966 KB)

Supplementary material 4 (AVI 4172 KB)

Supplementary material 5 (AVI 4327 KB)

Supplementary material 6 (AVI 5194 KB)

Supplementary material 7 (AVI 5308 KB)

Supplementary material 8 (AVI 5662 KB)
